# Visual Performance of a Violet-Filtering Intraocular Lens versus a Blue-Filtering Intraocular Lens of New/Old Manufacturing Processes

**DOI:** 10.3390/jcm12031195

**Published:** 2023-02-02

**Authors:** Santaro Noguchi, Shunsuke Nakakura, Asuka Noguchi, Hitoshi Tabuchi

**Affiliations:** 1 Department of Ophthalmology, Saneikai Tsukazaki Hospital, Himeji 671-1227, Japan; 2 ASUCA Eye Clinic Sendai Mark One, Sendai 980-0011, Japan

**Keywords:** intraocular lenses, violet light-filtering colored lens, area under log contrast sensitivity function, visual function, quality of life

## Abstract

This article compares the visual performance of a violet light-filtering colored lens (ZCB00V) and blue light-filtering intraocular lens (IOL; SN60WF) before and after modifying the manufacturing process for glistening suppression. In this retrospective study, conducted at Saneikai Tsukazaki Hospital, Himeji, Japan, a consecutive sample of 8943 eyes of 5119 patients were included and implanted with blue-filtering IOLs before and after modifying the manufacturing process (SN60WF-J (1318 eyes) and SN60WF-Q,A (1418 eyes), respectively), noncolored UV-cut IOLs (ZCB00 (1418 eyes)), and ZCB00V (3717 eyes). For each patient, the corrected distance visual acuity (CDVA) at 3 months postoperative (3MCDVA) and the area under log contrast sensitivity function (AULCSF) were measured. The 3MCDVA was −0.076 ± 0.1, −0.11 ± 0.13, −0.10 ± 0.17, and −0.11 ± 0.13, for SN60WF-J, SN60WF-Q,A, ZCB00, and ZCB00V, respectively. The SN60WF-J group revealed significant differences as compared to the other three groups (all *p* < 0.05). The mesopic AULCSF was 2.59 ± 0.20, 2.68 ± 0.19, −2.69 ± 0.18, and 2.76 ± 0.19, respectively, whereas the photopic AULSCF was 2.63 ± 0.23, 2.76 ± 0.25, −2.77 ± 0.25, and 2.88 ± 0.25. The SN60WF-J and ZCB00V groups exhibited significant differences as compared to the other three groups, whereas no significant differences were noted between the SN60WF-Q,A and the ZCB00 groups (all *p* < 0.05). The violet-filtering lens offers higher visual acuity and contrast sensitivity than the clear and blue-filtering lens. It was also found that the above functions were improved by modifying the manufacturing process.

## 1. Introduction

Among foldable intraocular lenses (IOLs) made of hydrophobic acrylic resin, Acrysof (Alcon Laboratories Inc.) is reported to be susceptible to light dispersion by accumulation of tiny vacuoles within the lens optic due to glistening after intraocular implantation [1,2,3,4]. Depending on the lens material, the degree of light dispersion differs among hydrophobic IOLs. The longer an implanted IOL stays within the eye, the greater is the increase in light dispersion [2,4]. Although corrected visual acuity reportedly decreases because of strong IOL glistening [4], other reports state that IOL glistening scarcely causes a decrease in visual acuity [2,3,4]. However, in an experiment using model eyes, the authors reported a decrease in contrast sensitivity [5]. The examination of glistening Acrysof IOLs that were extracted revealed that IOL glistening was the result of a phase separation of nanometer-sized water aggregates [6,7]. Based on these findings, the manufacturer implemented a reduction in humidity and strict temperature control during the Acrysof manufacturing process and tightened the process control and specifications for the combination, cast forming, and hardening procedures, thereby achieving optimization [8,9]. The line of Acrysof manufactured by the conventional process is named “J-code,” whereas the line manufactured by the new process is categorized as “Q-code.” Furthermore, an additional new line of Acrysof was developed as “A-code,” in which a risk of adhesion of potential foreign objects to the IOL is mitigated through a modification in the casting mold.

In contrast, among the TECNIS (Johnson & Johnson, New Brunswick, NJ, USA) line of hydrophobic acrylic resin IOLs [2,4], only clear IOLs were previously commercially available. However, the same manufacturer began marketing an IOL that adopted yellow-colored Optiblue glass, which filters only ultraviolet and violet light rather than filtering blue light that affects the circadian rhythm [10]. Acrysof Q-code, Acrysof A-code, and Optiblue IOL are all exclusively available or mainly available in Japan.

This study aimed to investigate how visual acuity and contrast sensitivity differ among the one-piece aspheric hydrophobic acrylic monofocal IOLs: Acrysof-material blue-filtering IOLs (SN60WF J-code, Q-code, and A-code), TECNIS-material clear IOL (ZCB00, Johnson & Johnson Vision Inc.), and Optiblue violet-filtering IOL (ZCB00V, Johnson & Johnson Vision Inc.). SN60WF (Blue Light Cut IOL) attenuates blue light emission (440–500 nm) and shorter wavelength light emission. In contrast, ZCB00V (violet-light-cut IOL) attenuates the most harmful component of visible light, the violet wavelength (400–440 nm) [10].

## 2. Materials and Methods

### 2.1. Patients

This study was approved by the Institutional Review Board of Saneikai Tsukazaki Hospital (Approval No. 211014), and the procedures followed were in accordance with the 1975 Declaration of Helsinki and its later amendments. This retrospective study included patients who underwent a cataract operation at Saneikai Tsukazaki Hospital between August 2009 and October 2020. Subjects were selected from among patients with no ocular diseases other than cataract, who developed no ocular complications intra- or postoperatively and who had no after cataract postoperatively. Overall, 1318 eyes of 785 patients were implanted with SN60WF J-code (SN60WF-J), 2490 eyes of 1420 patients were implanted with either SN60WF Q-code or A-code (SN60WF-Q,A), 1418 eyes of 816 patients were implanted with ZCB00, and 3717 eyes of 2098 patients were implanted with ZCB00V.

**Figure 1:**
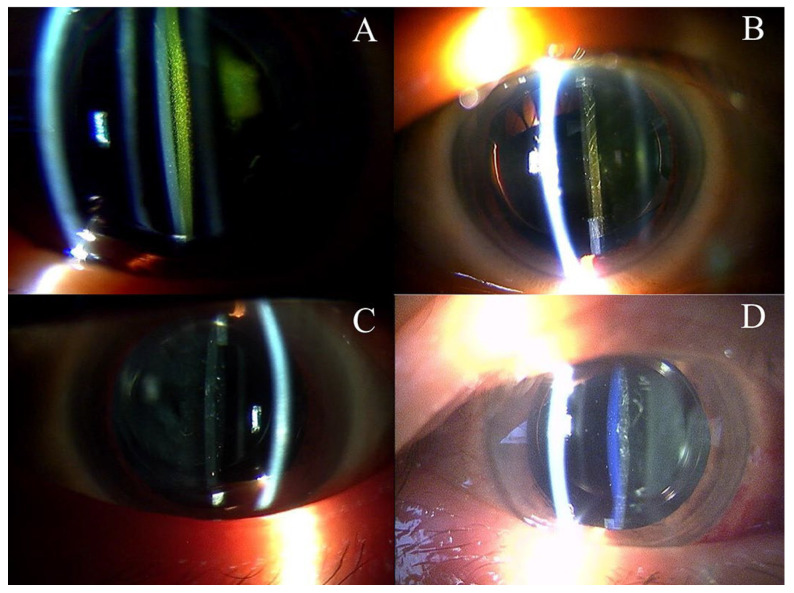
(**A**): Slit lump photograph of SN60WF J-code. (**B**): Slit lump photograph of SN60WF Q,A-code. (**C**): Slit lump photograph of ZCB00. (**D**): Slit lump photograph of ZCB00V.

SN60WF-J was implanted from August 2009 to December 2011. SN60WF Q-code was implanted from January 2012 to May 2016, and SN60WF A-code was implanted from June 2016 to October 2020. ZCB00 was implanted throughout the investigation period, whereas ZCB00V was implanted from January 2013 to October 2020 (Figure 1).

### 2.2. Examination Protocol

In all cases, the operation involved temporal corneal incision (2.2–2.4 mm) and phacoemulsification. As for pre- and postoperative examinations, patients were tested in the hospital’s outpatient department. Preoperative measurements included ocular axial length, flat and steep keratometry (Kf, Ks) (IOL Master^®^ 500, Carl Zeiss, Jena, Germany), uncorrected distance visual acuity (UDVA) 3 months postoperatively, and corrected distance visual acuity (CDVA) 1 week and 3 months postoperatively (1WCDVA and 3MCDVA, respectively; LC-10, Takagi Seiko, Nagano, Japan). Cumulative percentages were calculated for cases with a logarithm of the minimum angle of resolution (LogMAR) visual acuity of ≤−0.1, ≤0, 0, ≤1, and ≤0.2. Contrast sensitivity was measured 3 months postoperatively using a CGT-1000 device (CGT-1000, Takagi Seiko, Nagano, Japan) for six target sizes (1.1 cycles/degree (cpd), 1.8 cpd, 2.9 cpd, 4.5 cpd, 7.1 cpd, and 10.2 cpd) without glare (10 cd/m^2^) (mesopic condition) and with glare (40,000 cd/m^2^) (photopic condition). Based on the measurements, the area under log contrast sensitivity function (AULCSF) values (mesopic AULCSF and photopic AULCSF) were calculated. Moreover, the implanted IOL power and the manifest refractive spherical equivalent (MRSE) 1 week and 3 months postoperatively (1WMRSE and 3MMRSE) were also calculated. The total high-order aberration (HOA) was also measured for the entire globe aberration with Wave front analyzer KR-1W (TOPCON, Tokyo, Japan) 3 months postoperatively.

The data were expressed as mean ± standard deviation (SD) (range). Three months after the operation, some patients were not visited for the postoperative examination of the visual acuity test, contrast sensitivity test, subjective refraction test, and higher-order aberration test. Therefore, we analyzed the results three months after surgery for 810 eyes for SN60WF-J, 1355 eyes for SN60WF-Q,A, 903 eyes for ZCB00, and 2350 eyes for ZCB00V.

### 2.3. Statistical Analysis

For statistical analysis, we used JMP14.3 software and performed the Tukey–Kramer test, with the significance level set at *p* < 0.05. We retrospectively estimated a sample size requirement of 1006 for ZCB00V CDVA (LogMAR) to detect a 0.02 (1 log MAR letter) difference between the groups, with a significance level of 5% and power of 80%, according to an SD of 0.16 for the average of ZCB00V CDVA.

## 3. Results

We observed significant differences between certain groups in the preoperative axial length, implanted IOL power, Kf, Ks, and MRSE. However, no group was significantly aberrant. The total HOA for the entire globe did not show any significant intergroup differences (Table 1).

### 3.1. Visual Acuity Outcomes

**Figure 2:**
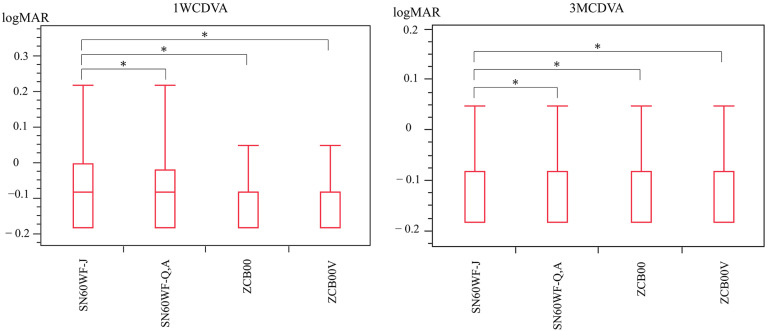
A comparison of corrected distance visual acuity 1 week postoperatively (1WCDVA, LogMAR) between the four groups shows that the SN60WF-J group had significantly higher and poorer visual acuity values than the other three groups did. Three months postoperatively (3MCDVA, LogMAR), the corrected distance visual acuity in the SN60WF-J group was significantly poorer than that of the other three groups. * Tukey–Kramer test, *p* < 0.05.

The 1WCDVA was −0.060 ± 0.18 in the SN60WF-J group, −0.085 ± 0.14 in the SN60WF-Q,A group, −0.096 ± 0.18 in the ZCB00 group, and −0.093 ± 0.16 in the ZCB00V group. The SN60WF-J group showed statistically significant differences as compared to the SN60WF-Q,A, ZCB00, and ZCB00V groups (all *p* < 0.05; Figure 2 left). The 3MCDVA was −0.076 ± 0.17 in the SN60WF-J group, −0.11 ± 0.13 in the SN60WF-Q,A group, −0.10 ± 0.10 in the ZCB00 group, and −0.11 ± 0.13 in the ZCB00V group. With regard to 1WCDVA, the SN60WF-J group showed statistically significant differences as compared to the SN60WF-Q,A, ZCB00, and ZCB00V groups (all *p* < 0.05; Figure 2 right).

**Figure 3:**
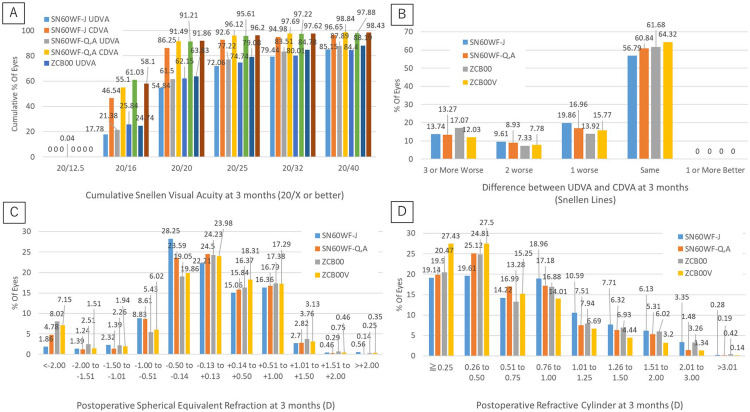
(**A**) Cumulative Snellen binocular acuity at 3 months (20/× or better). (**B**) Difference between uncorrected (UDVA) and corrected (CDVA) distance visual acuity at 3 months (Snellen Lines). (**C**) Postoperative spherical equivalent (diopters (D)) at 3 months. (**D**) Refractive cylinder (D) at 3 months.

With regard to the cumulative rate of visual acuity at 3 moths, for UDVA, the rate of Snellen visual acuity 20/20 was 54.84% in the SN60WF-J group, 61.50% in the SN60WF-Q,A group, 62.15% in the ZCB00 group, and 63.83% in the ZCB00V group. For CDVA, the rate of Snellen visual acuity 20/20 was 86.25% in the SN60WF-J group, 91.49% in the SN60WF-Q,A group, 91.21% in the ZCB00 group, and 91.86% in the ZCB00V group (Figure 3A).

### 3.2. Contrast Sensitivity Outcomes

In the SN60WF-J group, the mesopic contrast sensitivity was 1.54 ± 0.26, 1.43 ± 0.26, 1.32 ± 0.27, 1.11 ± 0.28, 0.81 ± 0.28, and 0.50 ± 0.20 (at 1.1 cpd, 1.8 cpd, 2.9 cpd, 4.5 cpd, 7.1 cpd, and 10.2 cpd, respectively). In the SN60WF-Q,A group, it was 1.6 ± 0.25, 1.55 ± 0.25, 1.44 ± 0.25, 1.24 ± 0.26, 0.95 ± 0.26, and 0.6 ± 0.22, respectively. In the ZCB00 group, it was 1.61 ± 0.23, 1.55 ± 0.24, 1.45 ± 0.24, 1.26 ± 0.26, 0.97 ± 0.26, and 0.63 ± 0.22, respectively. In the ZCB00V group, it was 1.67 ± 0.24, 1.63 ± 0.24, 1.53 ± 0.25, 1.33 ± 0.26, 1.05 ± 0.27, and 0.69 ± 0.25, respectively. Within 1.1 cpd to 7.1 cpd, we noted statistical differences between the SN60WF-J group and the other three groups, between the SN60WF-Q,A and ZCB00V groups, and between the ZCB00 and ZCB00V groups (all *p* < 0.05). At 10.2 cpd, all groups showed statistically significant differences from all the other groups (*p* < 0.05).

**Figure 4:**
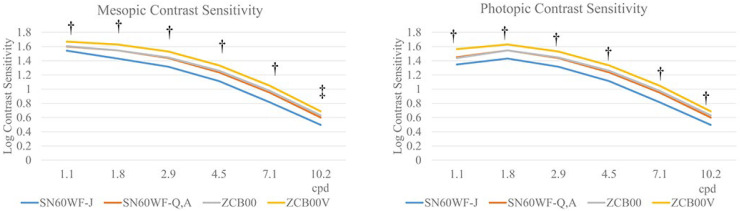
The mesopic contrast sensitivity of the SN60WF-J group was lower than that of the other groups at all cpds. Conversely, the ZCB00V group had significantly better contrast sensitivity. Although significant differences were observed between the SN60WF-Q,A and ZCB00 groups at 10.2 cpd (*p* < 0.05), no significant differences were observed at any other cpd. As in the mesopic condition, the contrast sensitivity in the SN60WF-J was lower at all cpd, whereas that in the ZCB00V was better. No significant differences were observed between the SN60WF-Q,A and ZCB00 groups at any cpd. There were ^†^ statistical differences between the SN60WF-J group and all other three groups, between the SN60WF-Q,A and ZCB00V groups, and between the ZCB00 and ZCB00V groups. At 10.2 cpd, all groups showed statistically significant differences from all the other groups (*p* < 0.05). ^‡^ All groups showed statistically significant differences from all the other groups (*p* < 0.05). Tukey–Kramer test, *p* < 0.05.

In the SN60WF-J group, the photopic contrast sensitivity was 1.35 ± 0.26, 1.43 ± 0.26, 1.32 ± 0.27, 1.11 ± 0.28, 0.81 ± 0.28, and 0.50 ± 0.20, respectively. In the SN60WF-Q,A group, it was 1.45 ± 0.28, 1.55 ± 0.25, 1.44 ± 0.25, 1.24 ± 0.26, 0.95 ± 0.26, and 0.60 ± 0.22, respectively. In the ZCB00 group, it was 1.44 ± 0.27, 1.55 ± 0.24, 1.45 ± 0.24, 1.26 ± 0.26, 0.97 ± 0.26, and 0.63 ± 0.22, respectively. In the ZCB00V group, it was 1.56 ± 0.28, 1.63 ± 0.24, 1.53 ± 0.25, 1.33 ± 0.26, 1.05 ± 0.27, and 0.69 ± 0.25, respectively. Within 1.1 cpd–10.2 cpd, we noted statistical differences between the SN60WF-J group and the other three groups, between the SN60WF-Q,A and ZCB00V groups, and between the ZCB00 and ZCB00V groups (all *p* < 0.05; Figure 4).

**Figure 5:**
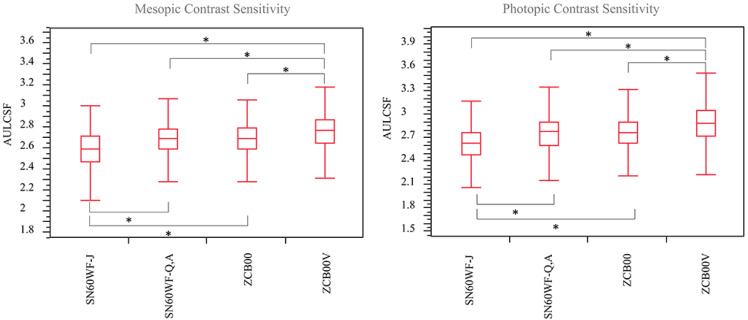
With regard to contrast sensitivity, the mesopic AULCSF were significantly poorer in the SN60WF-J group than in the other three groups. The ZCB00V group showed significantly higher values than the other three groups. No significant differences were observed between the SN60WF-Q,A and ZCB00 groups. * Tukey–Kramer test, *p* < 0.05.

The mesopic AULCSF was 2.59 ± 0.20 in the SN60WF-J group, 2.68 ± 0.19 in the SN60WF-Q,A group, −2.69 ± 0.18 in the ZCB00 group, and 2.76 ± 0.19 in the ZCB00V group (SN60WF-J vs. SN60WF-Q,A *p* < 0.001, SN60WF-J vs. ZCB00 *p* < 0.001, SN60WF-J vs. ZCB00V *p* < 0.001, SN60WF-Q,A vs. ZCB00 *p* = 0.58, SN60WF-Q,A vs. ZCB00V *p* < 0.001, and ZCB00 vs. ZCB00V *p* < 0.001). The photopic AULCSF was 2.63 ± 0.23 in the SN60WF-J group, 2.76 ± 0.25 in the SN60WF-Q,A group, 2.77 ± 0.25 in the ZCB00 group, and 2.88 ± 0.25 in the ZCB00V group (SN60WF-J vs. SN60WF-Q,A *p* < 0.001, SN60WF-J vs. ZCB00 *p* < 0.001, SN60WF-J vs. ZCB00V *p* < 0.001, SN60WF-Q,A vs. ZCB00 *p* = 0.98, SN60WF-Q,A vs. ZCB00V *p* < 0.001, and ZCB00 vs. ZCB00V *p* < 0.001; Figure 5).

## 4. Discussion

This study compared the visual functions of SN60WF before and after the manufacturing process modification, ZCB00, and ZCB00V. The 1WCDVA was significantly poorer in the SN60WF-J group than in the other three groups. Likewise, the 3MCDVA was also significantly poorer in the SN60WF-J group than in the other three groups.

The best contrast sensitivity at mesopic condition was observed in the ZCB00V group other than 10.2 cpd. Meanwhile, the contrast sensitivity was comparable between the SN60WF-Q,A and ZCB00 groups, and the SN60WF-J group exhibited the poorest contrast sensitivity. Under the photopic condition, intergroup differences in contrast sensitivity were larger. At lower cpd, the intergroup differences were larger, making it easier to observe differences in the lens properties. The mesopic and AULCSFs were significantly lower in the SN60WF-J group than in the other groups. Meanwhile, the values were comparable between the SN60WF-Q,A and ZCB00 groups. Compared with all the other groups, the ZCB00V group had significantly better contrast sensitivity. The photopic condition is not conducive to visual function measurement because of the bright and backlit environment. However, compared to that mesopic condition, the lens properties might have been clearer in photopic condition.

The SN60WF-J and SN60WF-Q,A groups use lenses made of an identical material, platform, spherical aberration, and color. Therefore, we inferred that differences in visual functions between these two variations may represent only the effects of the modification in the manufacturing process. Although a prior report confirmed the surface dispersion and glistening of Acrysof IOLs 1 to 2 years postoperatively [2], the corrected visual acuity 1 week postoperatively in this study was significantly poorer in the SN60WF-J group than in the other groups, including the SN60WF-Q,A group. These results suggest that the glistening that affects visual functions may occur as early as 1 week postoperatively. This tendency was unchanged 3 months postoperatively. Furthermore, the contrast sensitivity was significantly lower in the SN60WF-J group than in the SN60WF-Q,A group. Therefore, the modified manufacturing process for suppressing glistening was also effective for improving contrast sensitivity and corrected visual acuity.

As compared with the ZCB00, the SN60WF-Q,A consists of a different material, platform, spherical aberration, and color. Nevertheless, it was shown that SN60WF-Q,A performance was comparable to that of the ZCB00 in terms of contrast sensitivity.

Moreover, although ZCB00V has the same basic platform as ZCB00, except for the color, ZCB00V had significantly better contrast sensitivity. These results suggest that violet-filtering colored IOLs may be more conducive to improving contrast sensitivity than clear colored IOL. Furthermore, compared with blue-filtering colored IOLs, violet-filtering colored IOLs may have better contrast sensitivity. In particular, despite the differences in the lens materials and platforms, the spectral transmittance of the blue light-filtering SN60WF was 67% for violet light and 27% for blue light [10]. However, the violet-filtering ZCB00V blocks 90% of violet light and 6% of blue light [10]. Given these properties, violet-filtering color may be more beneficial for visual functions than blue light-filtering color. Nakano et al., reported that the contrast sensitivity of ZCB00V was better than that of SN60WF. This result partly corroborates our findings [11]. Moreover, Zigman et al. [12] reported that short-wavelength illumination augments light dispersion, chromatic aberration, and fluorescence. They argued that the contrast sensitivity and brightness of video footage improved both macroscopically and in mechanical images when wavelengths less than 450 nm were removed using cutoff filters, which is consistent with the findings of this study. Violet light (400–450 nm) is reported to be less important for vision, but has higher phototoxicity than blue light. There has been debate as to whether IOLs should transmit blue light for scotopic vision and circadian photoreception. Appropriately timed responses to light exposure have been reported to aid in effective circadian entrainment and good management of diurnal physiological bioresponses. The spectral sensitivity of circadian photoreception peaks at blue light in the spectrum around 460 nm. Inadequate blue light enhances insomnia, depression, and more [13].

If SN60WF-J is replaced with SN60WF-Q,A, a considerable improvement in visual functions is expected. ZCB00V is a violet-filtering colored single-focus IOL, which is also marketed exclusively in Japan. If this product is applied widely to various multifocal IOLs, patients may be able to acquire higher visual functions.

The supply of SN60WF-J was discontinued in December 2011 and replaced with the SN60WF-Q line of Acrysof IQ IOL in the following month. Meanwhile, ZCB00V was launched in January 2013. Because these IOL products have different marketing periods, conducting a comparison among patients within a completely identical time frame is impracticable. Accordingly, the effects of this mismatch might not be completely ruled out. Moreover, given the nature of retrospective studies, diseases other than cataract might have been overlooked. Furthermore, there were some statistical differences between the groups in patient backgrounds, such as age and ocular axial length. It is impossible to rule out the possibility that these factors affected the results of this study.

By changing the manufacturing process of SN60WF, the Acrysof IQ blue light-filtering material, to suppress glistening, contrast sensitivity and CDVA were improved, and the visual function became equivalent to that of the uncolored ZCB00. However, ZCB00V, the violet-filtered version of ZCB00, provided higher contrast and better visual function than ZCB00 and the revised SN60WF.

## Figures and Tables

**Table 1:** Backgrounds and characteristics of patients in the four groups ^a^.

	SN60WF-J	SN60WF-Q, A	ZCB00	ZCB00V	*p* Value					
					SN60WF-J vs. SN60WF-Q, A	SN60WF-J vs. ZCB00	SN60WF-J vs. ZCB00V	SN60WF-Q, A vs. ZCB00	SN60WF-Q, A vs. ZCB00V	ZCB00 vs. ZCB00V
*n*	785	1420	816	2098						
Eye	1318	2490	1418	3717						
Age (years)	73.06 (7.82)	72.5 (7.93)	71.34 (7.89)	72.78 (8.24)	0.18	<0.0001 *	0.71	<0.0001 *	0.53	<0.0001 *
IOL power (D)	20.17 (3.48)	19.92 (3.61)	19.76 (4.23)	19.96 (3.99)	0.22	0.02 *	0.33	0.57	0.97	0.31
Axial length (mm)	23.54 (1.28)	23.74 (1.33)	23.91 (1.57)	23.91 (1.5)	0.0003 *	<0.0001 *	<0.0001 *	0.002 *	<0.0001 *	1
Kf (mm)	7.65 (0.28)	7.69 (0.27)	7.65 (0.26)	7.7 (0.27)	0.0004 *	1	<0.0001 *	0.0004 *	0.55	<0.0001 *
Ks (mm)	7.51 (0.28)	7.51 (0.28)	7.54 (0.26)	7.53 (0.27)	1	0.07	0.05	0.05	0.02 *	0.99
1WMRSE (D)	−0.13 (0.76)	−0.15 (0.85)	−0.22 (1.06)	−0.19 (1.01)	0.95	0.07	0.28	0.11	0.43	0.71
3MMRSE (D)	−0.08 (0.73)	−0.15 (0.86)	−0.23 (1.08)	−0.18 (1.02)	0.23	0.002 *	0.03 *	0.13	0.76	0.46
Total HOA (4 mm)	0.14 (0.15)	0.14 (0.15)	0.14 (0.15)	0.14 (0.17)	0.53	0.66	0.39	1	1	1

Abbreviations: 1WMRSE, manifest refractive spherical equivalent at 1 week; 3MMRSE, manifest refractive spherical equivalent at 3 weeks; HOA, high-order aberration; IOL, intraocular lens; Kf, flat keratometry; Ks, steep keratometry. ^a^ Significant differences in age and ocular axis Kf were observed between some of the four groups. However, none of the four groups exhibited greatly aberrant mean values. * Tukey–Kramer test, *p* < 0.05.

## Data Availability

The data that support the findings of this study are available from the corresponding author upon reasonable request.
